# Sexual exploitation of male youth: background characteristics and needs from a life-course perspective

**DOI:** 10.1192/j.eurpsy.2024.536

**Published:** 2024-08-27

**Authors:** G. Mercera, S. Leijdesdorff, E. Heynen, T. van Amelsvoort

**Affiliations:** ^1^Psychiatry and Neuropsychology, Maastricht University, Maastricht; ^2^Clinical Psychology, Open University, Heerlen, Netherlands

## Abstract

**Introduction:**

Sexual exploitation is a human rights violation that has a detrimental impact on the psychological-, physical- and social well-being of victims. Sexually exploited male youth are prevalent, yet underrepresented in clinical practice, policy and research. There are multiple barriers that often prevent male youth to disclose and to seek or receive support, such as gender norms, limited awareness of victimization and feelings of guilt and shame.

**Objectives:**

By gaining more insight into the background and clinical characteristics of male victims and their care and support needs, this study aims to raise awareness and to better inform policymakers, care- and educational professionals on adequate prevention and intervention efforts.

**Methods:**

Twenty-six male youth at high-risk or victims of sexual exploitation participated in this qualitative study. By means of semi-structured interviews and case-file analyses, data was collected by to identify risk and protective factors in their life-course and care and support needs.

**Results:**

Results indicate that several vulnerabilities (e.g. previous experiences of abuse and neglect, mental health problems, household dysfunction, social rejection, running away, substance use) and a lack of positive and supportive relationships led male youth into high-risk situations. Among these were involvement in pay dates, survival sex and criminality, which contributed to victimization. Experiences of stigmatization were often a barrier to express vulnerabilities and to disclose victimization. There was a wide variety in care and support needs, including peer-to-peer support, therapy, support with day-to-day practices and anonymous support.

**Conclusions:**

These results will contribute to adequate prevention and intervention strategies and meet the unique needs of male youth at risk for, or victim of sexual exploitation.

**Disclosure of Interest:**

G. Mercera Grant / Research support from: This research was funded by the Dutch Ministry of Health, Welfare and Sports (grant number: 328604)., S. Leijdesdorff: None Declared, E. Heynen: None Declared, T. van Amelsvoort: None Declared

